# Perception of animacy leads to expectation of goal-directed behaviour in dogs

**DOI:** 10.1038/s41598-025-26837-w

**Published:** 2026-01-13

**Authors:** Zsuzsanna Gedai, Ádám Miklósi, Judit Abdai

**Affiliations:** 1 Department of Ethology, Eötvös Loránd University, Budapest, Hungary; 2 HUN-REN-ELTE Comparative Ethology Research Group, Budapest, Hungary; 3 Center for Mind/Brain Sciences, University of Trento, Rovereto, Italy

**Keywords:** Goal-directed behaviour, Goal attribution, Animacy perception, Robot, Dog, Expectancy violation, Anticipatory look, Animal behaviour, Social evolution, Social behaviour

## Abstract

Human newborns preferentially attend to self-propelled objects, and three-month-olds expect them to act in a goal-directed manner. Dogs also prefer animate objects, and anticipate human actions to be goal-driven, but it is unclear whether perceiving an object as animate leads to the expectation that it has goals. Here, first dogs observed either (1) a human moving around the room, (2) a robot interacting with a human, (3) a robot displaying self-propelled motion, or (4) a robot moving ambiguously. Next, the human or the robot repeatedly approached one of two objects. Finally, the objects’ places were switched, and the actor approached the same-object or same-location as before. We measured dogs’ look toward the objects after switching their locations. Dogs looked at the goal object before the actor turned toward either object, and looked longer at the goal object when the actor moved in a route-consistent, instead of a goal-consistent manner. These suggest that irrespectively of the actor type, dogs anticipated it to approach the goal-object, and the actor violated this expectation when moving to the same location. Thus, dogs’ goal attribution is not uniquely tied to human action, but it can be elicited by broader features of animacy.

**Supplementary Information:**

The online version contains supplementary material available at 10.1038/s41598-025-26837-w.

## Introduction

Human infants from birth seem to attend more to objects displaying simple animate motion cues, such as starting to move from rest^1^, showing sudden changes in speed^2^, and at least from six months of age, they rapidly encode the axial direction of a novel agent and use it to predict the direction of its action^3^ (no data available from younger infants). Core knowledge system suggests that human infants have innate, dedicated mechanisms distinguishing animate and inanimate objects, and their characteristics, such as the actions of animate agents are goal-directed and efficient^4^. The cue-based bootstrapping model^5^ proposes also the involvement of innate processes, but it suggests that the sensitivity to low-level motion cues provide a basis for further learning. The results of Di Giorgio et al.^2^ demonstrated that indeed, early sensitivity to animate cues (sign stimuli) directs infants’ attention to animate entities, and suggested that such innate biases to specific cues can facilitate further learning about animate-inanimate characteristics.

Not only that self-propelled motion alone seems to be sufficient to perceive an object as animate, but human infants also develop different expectations toward the actions of these agents compared to inanimate ones (e.g.^6,7^). Woodward^8^ developed a habituation-dishabituation paradigm to investigate infants’ understandings of goal-directed actions. Infants were habituated to a scene in which a human hand grasped the same toy out of two objects in repeated trials, which was always on the same side. They measured infants’ looking duration at the action until they reached habituation. This was followed by a dishabituation phase where the positions of the toys were switched, and the actor either grasped the same object on a novel route (goal-directed action) or used the same route toward a novel object. Infants from five months of age showed dishabituation only when a human actor switched its goal, but not when it used a novel route to reach the same goal. However, if instead of a human a rod was used as an actor, infants dishabituated when it used a new route. Thus, results indicated that infants attribute goal-directed actions to humans, but not to inanimate actors. Later, Sommerville et al.^9^ also showed that human infants as early as three months of age already expect a human action to be goal-directed (at least when they have direct experience with the task).

In the past two decades several researchers investigated whether human infants only expect humans to act in a goal-directed manner or perceiving an object as animate can lead to the expectation of having goals as well. Shimizu and Johnson^10^ applied a similar paradigm as Woodward^8^, and they found that by 12 months of age infants attribute goals to an unfamiliar agent if it displayed interactive behaviour earlier. Luo and Baillargeon^7^ also showed that infants as young as five months of age already attribute goals to a self-propelled agent, and at three months, if the agent shows self-propulsion, equifinality (i.e., using multiple routes to the same goal), and changes in the behaviour to reach the goal (trajectory direction)^6^. Using a different procedure, in Csibra^11^ a box used either one or two routes around an obstacle to reach an object. In the test phase, he removed the obstacle, and the box used either the same detour route or moved straight to the object (expected in case of goal-directed behaviour). His result demonstrated that this simple variability in the motion is enough for 6.5-month-old infants to consider the agent as driven by goals, even without an organism-shaped body. Note however, that the results of Biro et al.^12^ suggest that although self-propulsion may generate expectations about the goal-directed behaviour of an animate object, it rather biases these interpretations than serving as a prerequisite.

Several influential frameworks have been put forward to account for how human infants attribute goals to others’ actions, such as the teleological stance^13^, core knowledge of agency^4^, or cue-based bootstrapping^5^ (see also goal-directed imitation theory^14^). While our study does not aim to investigate the cognitive mechanism underlying goal-attribution, testing whether non-human animals also attribute goals to animate objects provides important comparative insight into the evolutionary background of this capacity.

Studies suggest that apes (bonobos, *Pan paniscus*; chimpanzees, *Pan troglodytes*; orangutans, *Pongo abelii*)^15^ view the actions of humans as goal-directed as well, and common marmosets (*Callithrix jacchus*) process the actions of humans, conspecific and a monkey-like robot as goal-directed, in contrast to inanimate objects^16,17^. Thus, it seems that non-human primates also expect goal-oriented motion from an agent, at least when it has physical characteristics resembling animals (e.g. face, legs). However, it is unclear how embodiment and specific motion cues contribute to such attribution in non-human species.

In dogs (*Canis familiaris*), Marshall-Pescini et al.^18^ applied a habituation-dishabituation paradigm (see^8^). Their results showed similar behaviour in dogs as in human infants, that is, dogs looked longer at the new-goal of the actor than at the new-route trials in the case of a human, but not when the actor was an inanimate object (black box moved by the experimenter from behind a curtain, using a long stick). However, recently Lonardo et al.^19^ found different results regarding the attribution of goal-directedness to humans. Dogs observing videos of a human hand vs. mechanical claw reaching for one of two objects, or a human vs. an inanimate actor approaching and kicking an object, Lonardo et al.^19^ found that anticipatory look does not suggest dogs expecting either a human or an inanimate actor to keep approaching the same (goal) object. Based on the changes in dogs’ pupil size and the latency to anticipatory fixation, dogs seemed to be more likely to expect both actors to approach the same location as before. Although, in one of their experiments, dogs fixated sooner on the previous side of the goal-object when the actor was an inanimate object than in the case of a human. Lonardo et al.^19^ proposed that dogs not having motor experience with grasping or kicking an object might cause the lack of anticipation of the specific action (for the importance of motor experience with the observed action in human infants, see also e.g.^9,20^), and that the human actor might divert dogs’ attention from the target of the action thus focusing on the location rather than on the object.

Previous studies suggest that dogs, similarly to human infants, perceive an object as animate based on simple motion cues. Dogs not only preferentially approached and interacted with an artificial agent (Unidentified Moving Object, UMO) that previously participated in a chasing event over those moving independently from each other^21^, but they also looked at and touched more frequently a UMO previously displaying animate motion (starting to move from rest and having changes in the speed) than a UMO with ambiguous motion^22^. Further, studies investigating dog-UMO interactions found that simple actions, such as, variability and reactivity in behaviour, and helping the dog to obtain an unreachable object can facilitate to establish social(-like) interaction between dogs and UMOs (e.g.^23–25^). Thus, it seems that self-propelled motion also attracts dogs’ attention to these objects, and that complex animate motion can facilitate dog-UMO interactions, but it is unclear whether animacy perception based on simple cues (e.g., self-propelledness alone) leads to further expectations regarding the behaviour of the objects, for example, their actions to be directed toward goals.

Here our aim was to test whether dogs attribute goal-directed motion to an unfamiliar object if it displays animate motion or interactive behaviour. We included four different actors, and in the Introduction phase, dogs observed the typical behaviour of one of them (between-subject design): (1) a human actor moving around the room with varying speed, stopping and turning a few times, to assess whether in our setup dogs expect human actions to be goal-directed (see^18^ vs^19^); (2) a UMO that engaged in an interaction with a human; (3) a UMO that showed motion cues associated with animacy (e.g., starting to move from rest, sudden changes in speed); and (4) a UMO displaying ambiguous motion, that is, the UMO appeared already in motion, moved with constant speed, and its stops and turns were not visible (a control stimulus often used in animacy perception studies, e.g.^2,26,27^).

Then, during the Demonstration phase, the actor repeatedly approached the same (goal) object out of two, across ten trials. In the Test phase, we switched the locations of the two objects, and the actor either approached the same goal object (three trials) or used the same route (three trials) (counterbalanced order). Although we applied a similar method as the classic habituation-dishabituation paradigm (e.g.^18^), we modified it in multiple ways to improve comparability of the human and UMO actors, and to be able to assess anticipatory look at the objects: (1) the actor stayed in the room during the entire experiment, (2) the actor did not physically engage with the objects, (3) at the beginning of the route, the actor moved forward straight (within barriers), (4) individual level habituation and dishabituation was not tested. Due to these changes (especially lack of individual habituation), we only measured subjects’ looking behaviour toward the objects in the Test phase: before the actor turned toward either object (anticipatory look), and after the actor turned toward one of them (violation of expectation).

We hypothesised that if dogs attribute goal-oriented behaviour to the specific actor, they look longer at the goal object before the actor turns toward it, anticipating its action, and also look longer at the goal object after the actor turns toward the non-goal object, because the actor’s action violates their expectation. We hypothesised that dogs anticipate not only the action of the human, but also that of the previously interactive and animate UMOs to be driven by a goal, because we expect dogs to hold different expectations toward animate and inanimate actors, and that such goal attribution is not restricted to humans but may depend on general cues of animacy. Alternatively, animate motion alone may not be sufficient for such attribution, and either more complex behaviour (interactivity) is needed for goal attribution, or a UMO, irrespectively of its behaviour, is not considered as a goal-directed agent by dogs.

## Results

### Looking at the objects in test trial 1 from start to turn (anticipatory look)

**Fig. 1:**
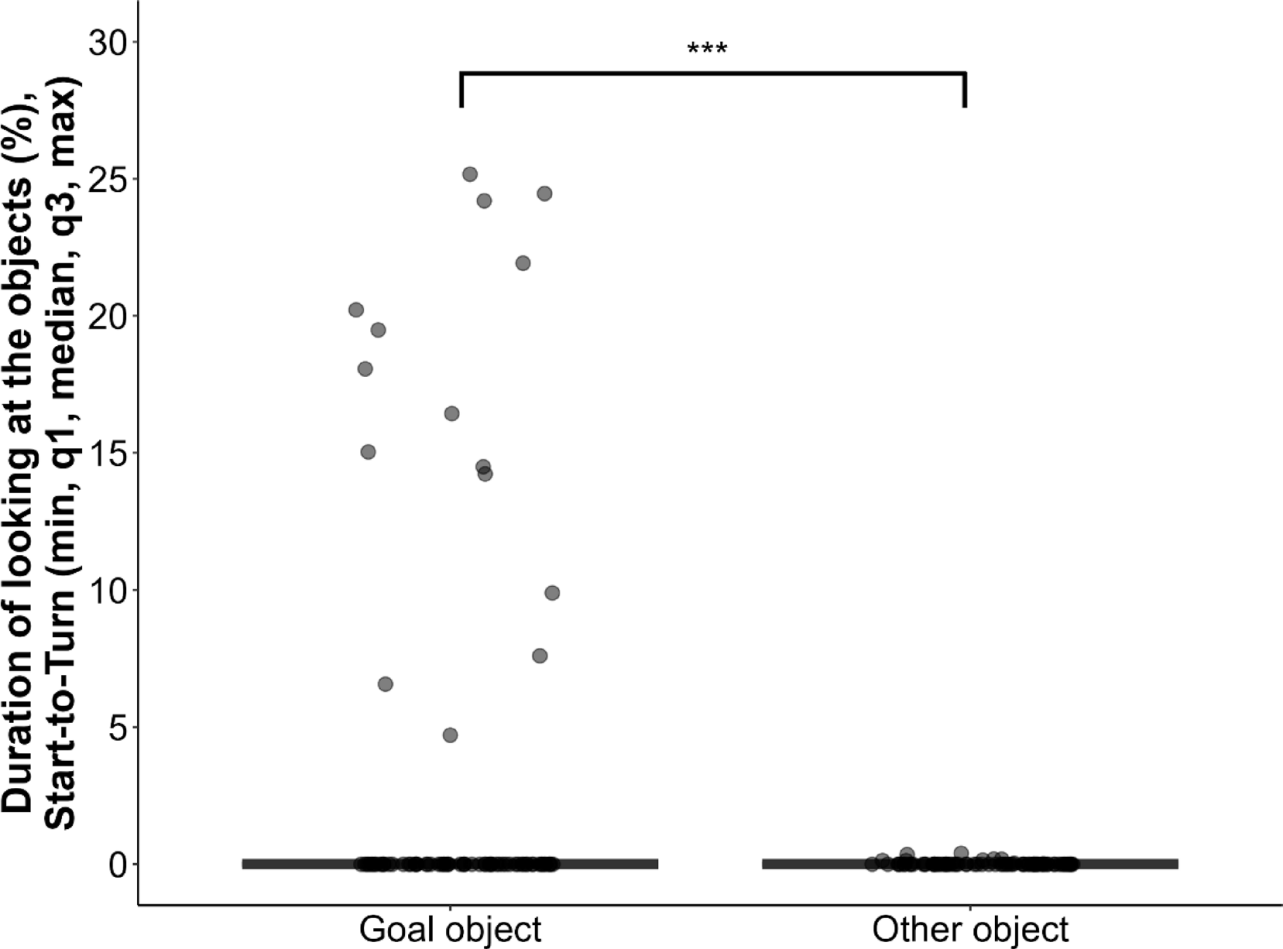
Percentage of looking at the goal and at the other object in Test trial 1, before the actor turned toward either of the objects (anticipatory look; Start-to-Turn). The figure depicts the percentage of looking at the objects; dogs looked longer at the goal than at the other object, irrespectively of the group. *** *p* < 0.001.

To see if dogs anticipate the actors to move toward the same object as before the objects’ locations were switched, we measured the percentage of their looking time toward each object, in the first trial of the Test phase (Test trial 1). Looking time was assessed after the actor started to move (Start) but before turning (Turn) to either of the objects (see Fig. 1), that is, when dogs could see the change in the object location, but the actor not yet engaged with either of them. We only assessed anticipatory look in this trial, because up to this point, the actor had always approached the same object at the same place. After switching the object positions, we could assess whether the dog expected the actor to move in a goal- or route-consistent way. However, in the following trials, the objects remained at the same locations as in Test Trial 1, and the dog had already seen the actor approach one of them. Thus, in these trials their anticipatory look could have been influenced by experience gained during Test trial 1 and so these trials were not included in the analysis.

**Fig. 2:**
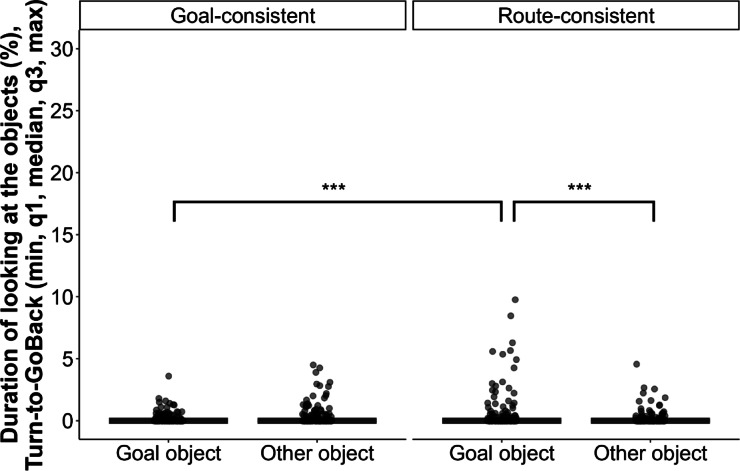
Percentage of looking at the goal and at the other object when the actor used a goal-consistent or route-consistent approach of the objects. Looking duration was measured from the moment the actor turned toward the object, until it started to move back to its original position (Turn-to-GoBack), assessed in Test trials 2, 3, 5, and 6. Group had no effect on the behaviour. *** *p* < 0.001.

We found that dogs looked longer at the goal object than at the other object (GLMM with zero-inflated tweedie distribution: Object, $\:{{\upchi\:}}_{1}^{2}$ = 59.856, *p* < 0.001; Goal vs. Other, β ± SE = 5.080 ± 0.507, *p* < 0.001), irrespectively of the group (Group x Object, $\:{{\upchi\:}}_{3}^{2}$ = 0.972, *p* = 0.808; Group, $\:{{\upchi\:}}_{3}^{2}$ = 2.386, *p* = 0.496) (Fig. 2).

### Looking at the objects in the test phase from turn to goback (violation of expectation)

We also tested whether dogs’ expectations were violated when the actor used the same route as before the switch in the object location (i.e., a route-consistent manner, approaching the other, non-goal object). We measured the percentage of dogs’ looking time toward each object, after the actor turned toward one of the objects (Turn), until it turned away and started to go back to its starting position (GoBack) (see Fig. 1). Looking time was assessed in Test trials 2–3 and Test trials 5–6, that is, the trials following the demonstration of the actor moving in a goal-consistent or route-consistent way (Test trials 1 and 4). We only used these trials, as Test Trial 1 followed the switch in the object position, likely leading to increased cognitive load due to novelty and making it difficult to isolate the violation of expectation responses. Test Trial 4 introduced a switch in the actor’s behaviour compared to the previous three trials, which could introduce additional uncertainty. By analysing Trials 2–3 and 5–6, we ensured that subjects had at least one experience with the actor’s behaviour in the new arrangement, allowing for more reliable detection of expectancy violation.

**Fig. 3:**
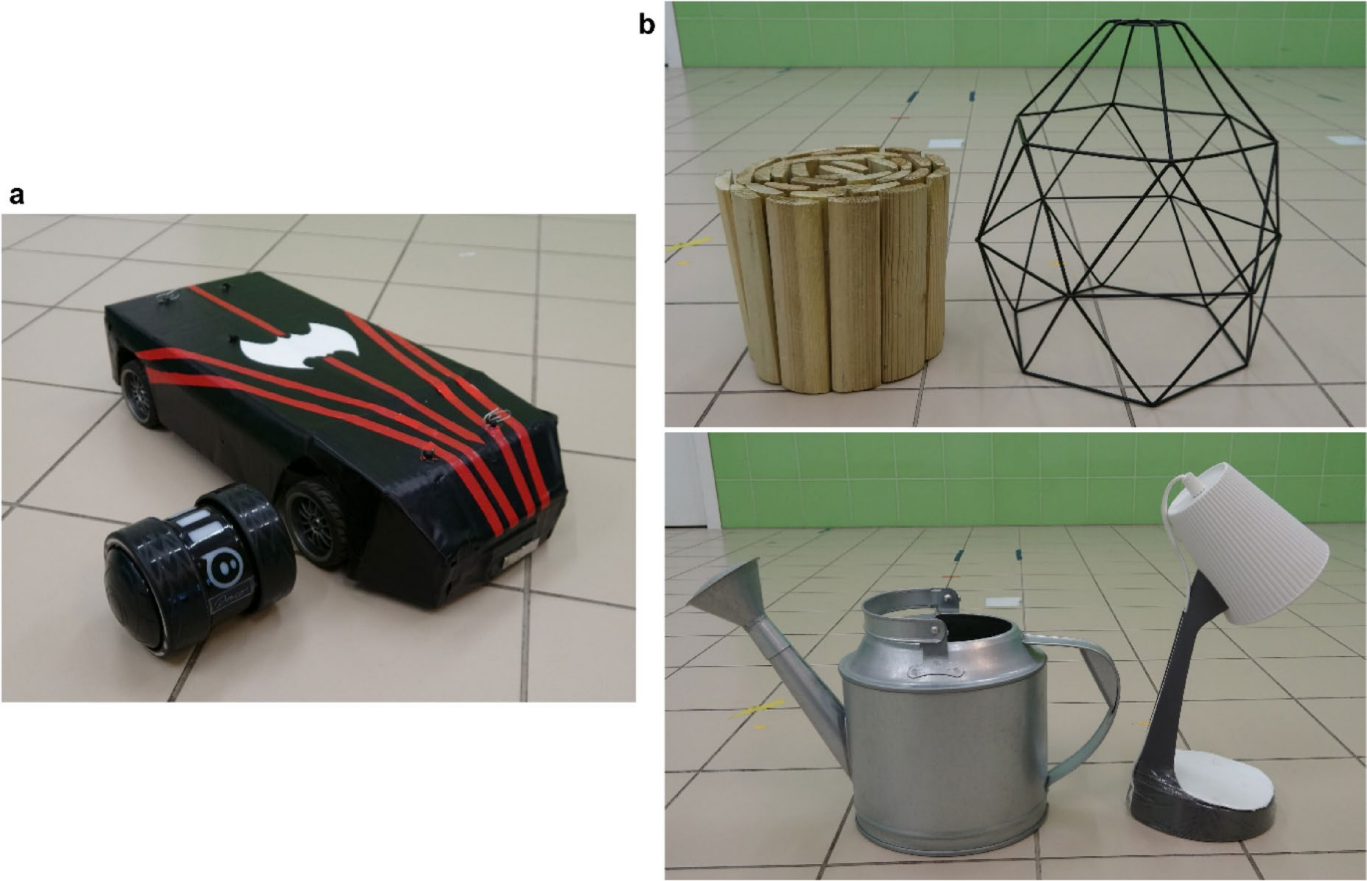
Experimental equipment and actors. (**a**) The two UMOs used as actors in the three UMO groups. (**b**) The two object pairs used in the Demonstration and Test phases.

We did not find significant difference between the groups regarding dogs’ looking time at the goal or at the other object (GLMM with zero-inflated tweedie distribution, LRT: Group x Section: $\:{{\upchi\:}}_{3}^{2}$ = 2.915, *p* = 0.405; Group x Object: $\:{{\upchi\:}}_{3}^{2}$ = 3.844, *p* = 0.279; Group: $\:{{\upchi\:}}_{3}^{2}$ = 2.577, *p* = 0.462). However, there were differences in dogs’ look at the objects depending on whether the actor approached the goal object or other, non-goal object (Object x Section, $\:{{\upchi\:}}_{1}^{2}$ = 12.429, *p* < 0.001) (Fig. 3). When the actor approached the other object using the same route as during the Demonstration phase, dogs looked at the goal object longer than at the other object (Route-consistent approach: Goal vs. Other object, β ± SE = 1.304 ± 0.357, *p* < 0.001). Further, dogs looked at the goal object longer when the actor approached the other object (Route-consistent) than when approaching the goal object (same object) (Goal object: Goal-consistent vs. Route-consistent approach, β ± SE = −1.677 ± 0.352, *p* < 0.001). However, when the actor approached the goal object, dogs did not look differently at the two objects (Goal-consistent approach: Goal vs. Other object, β ± SE = −0.562 ± 0.379, *p* = 0.139), and there was no difference between looking at the other object in the two sections (Other object: Goal-consistent vs. Route-consistent approach, β ± SE = 0.189 ± 0.370, *p* = 0.609).

Whether the goal or the route changed first in the Test phase did not influence subjects’ look at the objects (Change: $\:{{\upchi\:}}_{1}^{2}$ = 0.082, *p* = 0.775).

## Discussion

Our results show that following the repeated demonstration of a human or a UMO approaching the same object, dogs anticipated the actor to go to the same object even when it was at a new place, and they also looked at the goal object longer when the actor approached the other object (using its usual route). These results suggest that irrespectively of the type of actor, dogs recognised their action as goal-oriented. This result is in line with our hypotheses about the Human, Interactive UMO and Animate UMO groups, that is, dogs not only expect a regular social partner (human) to move in a goal-directed manner, but if a novel actor displays interactive behaviour or animate motion, dogs develop further expectations about their behaviour.

Dogs’ behaviour in the Ambiguous UMO group not differing from the other groups may seem unexpected. In previous studies with human infants (e.g.^6,7,11^; see also non-human primates^15,16^, and dogs^18^), researchers found that participants only attribute goals to humans or animate agents but expect inanimate actors to continue using their usual routes. Here, in the Introduction phase, the Ambiguous UMO did not show specifically inanimate, rather an ambiguous motion, that is, the UMO’s motion was not displayed as being caused by an external force, instead no information was provided about the onset of the motion. This ambiguity is sufficient in preference tests when contrasted with animate motion cues (see e.g., human infants^1^; chicks (*Gallus gallus*)^28^; dogs^22^; zebrafish (*Danio rerio)*^27^). But here, during the Demonstration and Test phases, the UMO displayed motion indicative of self-propulsion (starting to move from rest and changing motion direction without visible external cause). This could result in dogs perceiving this, previously inanimate/ambiguous UMO as animate as well. Biro et al.^12^ also suggested that if behavioural cues change over time in a way that they are in conflict with their initial categorization, humans change the categorization of the actor to match to the behavioural cues and make predictions about its future behaviour based on this novel category. To our knowledge, no studies have investigated how such categorization may change (from animate to inanimate, or vice versa) and how does it influence further expectations. However, such research could be important to shed light on how initial perception and novel information shapes expectations toward these actors.

In Lonardo et al.^19^, the moving, human-sized box seemed to be self-propelled (cf.^18^) which could be enough to perceive it as animate, and it not only approached, but repeatedly bumped into the object. Although they also did not find difference in dogs’ looking behaviour (including pupil dilation) toward the actions of the human or box, in their study this indicated the lack of goal-attribution to either actor. However, regarding the human actor, our results are consistent with the previous findings of Marshall-Pescini et al.^18^, that is, dogs expect a human to move toward a previously established goal. Differences in the experimental setups (e.g., live vs. video display, relying on habituation or not, or the action showed by the actor upon reaching the objects, etc.) can account for the differences in the three studies. Future research is needed to reveal how specific aspects of the methodological approach influences dogs’ behaviour in this situation. Relying on looking duration alone may not be conclusive about goal attribution, but applying complementary physiological measures, such as pupil dilation (e.g.^19,29,30^), can provide further insights and a more comprehensive assessment of the underlying processes.

Despite studies showing differences of goal-attribution to animate vs. inanimate actors (e.g.^6,7,18^), the results of Biro et al.^12^ provide a more nuanced picture. Their findings indicate that perceiving the actor as animate is not a prerequisite to attribute goal-directed motion to the object in 12-month-old human infants, rather it can facilitate to interpret the action as directed toward a goal. They proposed that inferring goal to an actor relies on the rationality of the action, that is, the actor acting efficiently toward a goal. However, in their scenario the actor’s action could be interpreted as goal-directed via the efficiency of the action (jumping over a barrier vs. jumping without a barrier present) whereas in the present study, efficiency/rationality in movement was not presented. As dogs in the Ambiguous UMO group showed similar behaviour as those in the other groups, we suggest that in future studies, a modified control group should be introduced, where the motion of the Ambiguous UMO is clearly inanimate (e.g., the subject observes its motion being initiated by external force). This would allow to disentangle whether it is animacy per se, or other aspects of the motion facilitate the attribution. In the current experimental setup, we were unable to have a fully inanimate, moving actor without introducing additional confounds (e.g., detours, external moving elements, or distracting apparatus). Specifically, occluding the onset of the UMO’s motion at the beginning of the route and when leaving the object would have required alterations that themselves could create salient cues, risking distraction. Further research is needed to assess in details how specific animate/social cues contribute to such attributions, and to explore alternative setups to be able to introduce stricter inanimate controls.

To summarize our findings, it seems that similarly to human infants (e.g.^6,7^), dogs recognize animate actors as goal-directed agents, and they not only have simple expectations toward the animate actors, such as having the ability to move without external force, but also anticipate more complex capacities such as its actions to be goal-directed. Although the lack of difference between the Interactive, Animate and Ambiguous UMO groups warrants caution regarding whether specifically animate motion cues are required, our results reveal that dogs’ attribution of goal is not uniquely tied to human action, instead it can be triggered by general cues of animacy. While the current study did not focus on whether dogs’ behaviour is consistent with specific theoretical models, the results may reflect shared evolutionary roots in cognitive systems of agency detection and provide a strong basis for future comparative research in the mechanisms underlying goal attribution.

## Methods

### Subjects

The National Animal Experimentation Ethics Committee provided ethical approval (PE/EA/00032 − 4/2023). All methods were carried out in accordance with relevant guidelines and regulations, the experiment was performed in accordance with the EU Directive 2010/63/EU and all methods are reported in accordance with the ARRIVE guidelines. Owners provided written consent indicating voluntarily allowing their dogs to participate in the study and that the test videos can be used for publication of identifying images in an online open-access publication. The individuals depicted in the visual materials (figure and video) are two of the authors of this manuscript, Zsuzsanna Gedai and Judit Abdai.

We included dogs of different breeds above one year of age that did not have any previous experience with remote controlled cars, robot vacuum cleaner or Sphero robots (no such requirement when the human was the actor). We tested 90 dogs, ten of which were excluded: 6 dogs due to technical problems (e.g., Sphero Ollie robot lost the Bluetooth signal, had malfunction in the program or the battery discharged), 2 dogs because they looked at the event in the Introduction phase in less than 20% of the time, 1 dog displayed distressed in the room, and 1 dog was distracted by noises from outside. We had 80 dogs in the final analyses; subjects were assigned to four different groups: *Ambiguous UMO group* (*N* = 20; 9 females, mean ± SD age: 5.2 ± 3.3 years), *Animate UMO group* (*N* = 20; 11 females; mean ± SD age: 6.1 ± 4.0 years), *Interactive UMO group* (*N* = 20; 9 females; mean ± SD age: 5.3 ± 3.4 years), and *Human actor group* (*N* = 20; 15 females; mean ± SD age: 3.6 ± 2.4 years) (for more details see Supplementary Data 1).

### Actors and apparatus

**Fig. 4:**
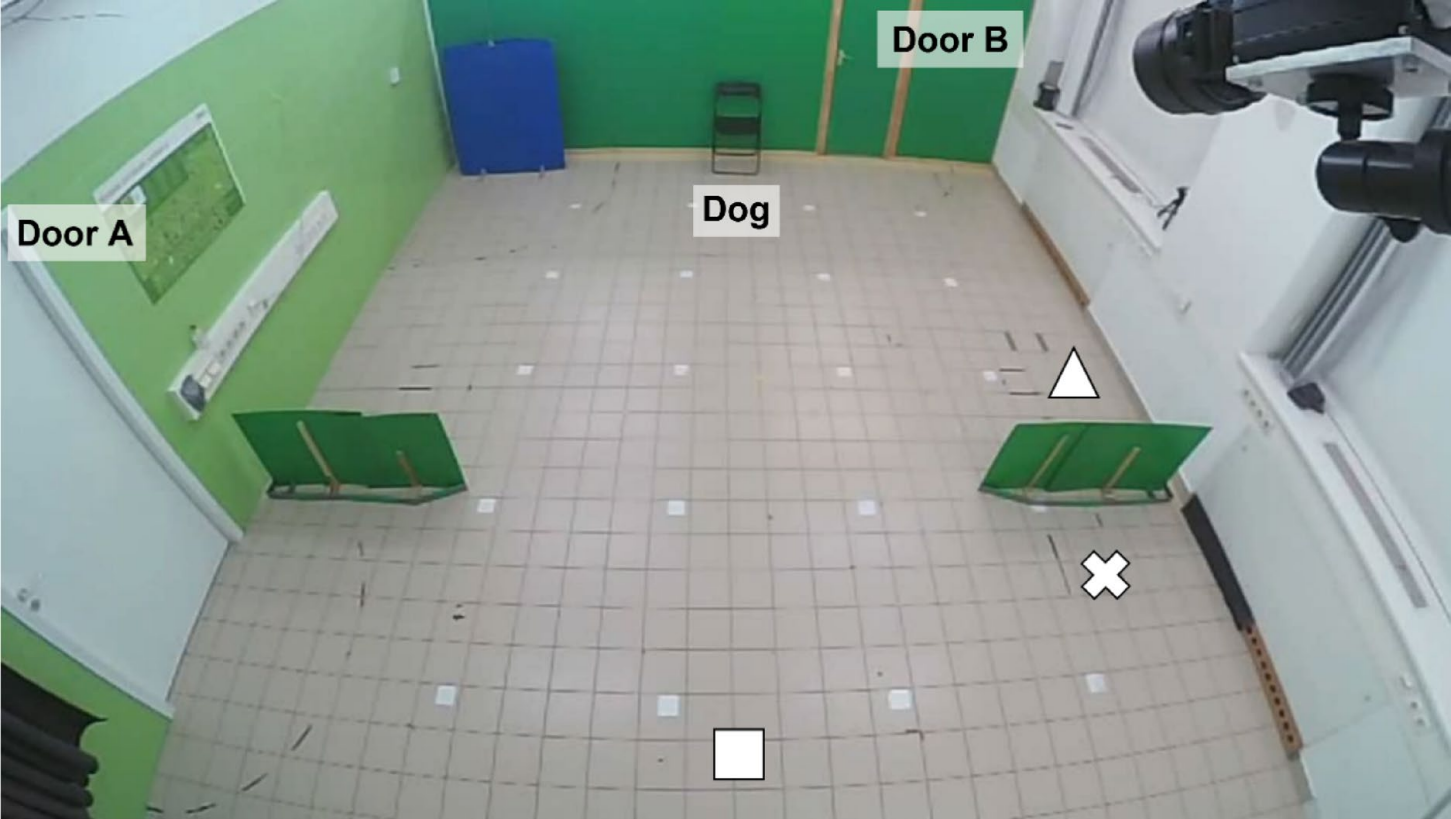
The arrangement of the room in the Introduction phase in the Ambiguous UMO group (the screens are only present here, in the other groups the room is empty except for the chair). Square: starting position of the Human and Interactive UMO; Triangle: starting position of the Animate UMO; Cross: starting position of the Ambiguous UMO.

In all three groups having a UMO as actor, we used two different UMOs to increase generalisability of our results for UMOs (Fig. 4). One of the UMOs was a remote-controlled car (#120090 E10 Michele Abbate GrrRacing Touring Car (L x W x H: 42 cm x 18 cm x 8.5 cm) with black cover. The other UMO was a Sphero Ollie Darkside (Sphero, Inc.; L x H: 11.94 cm x 8.13 cm) connected to the Sphero Edu app on an Android 8.0.0. smartphone via Bluetooth. In the Human actor group, the actor was an adult female human unfamiliar to the dog. We also had two different persons playing the human actor. The same dog only met one single type of actor; for half of the dogs one human, and the other half the other human, and for half the dogs in all UMO groups the RC car, and for the other half the Sphero Ollie was presented as an actor.

The remote-controlled car was controlled by the Experimenter (E) from outside through two wide-angle lens cameras (Zoom Q2n). The motion of the Sphero Ollie was pre-programmed, the UMO’s motion pattern was initiated by E on the smartphone that was in the room (close to the door used by E in the specific phase). E started the program before leaving the room. In the Introduction phase, there was a 12 s delay, in the Demonstration and Test phases a 20 s delay from the onset of the program until the first movement of the Ollie, giving enough time for E to leave the room and reach the camera room.

The test was carried out at the Department of Ethology, Eötvös Loránd University, Budapest, Hungary in a 6.27 m x 5.40 m room. Video recordings were made by three wide-angle lens cameras: two attached to the ceiling covering the entire room (Zoom Q2n) and one (Mobius ActionCam) on a tripod at the windowsill in front of the dog, focusing on the dog and having both objects in the frame.

Four objects (two pairs) were used as goal or non-goal objects during the Demonstration and Test phases: watering can and lamp or folded wooden fence and wire lamp shade (Fig. 4b). The object pairs, the side of the objects and which object was presented as the goal were counterbalanced between subjects.

**Fig. 5:**
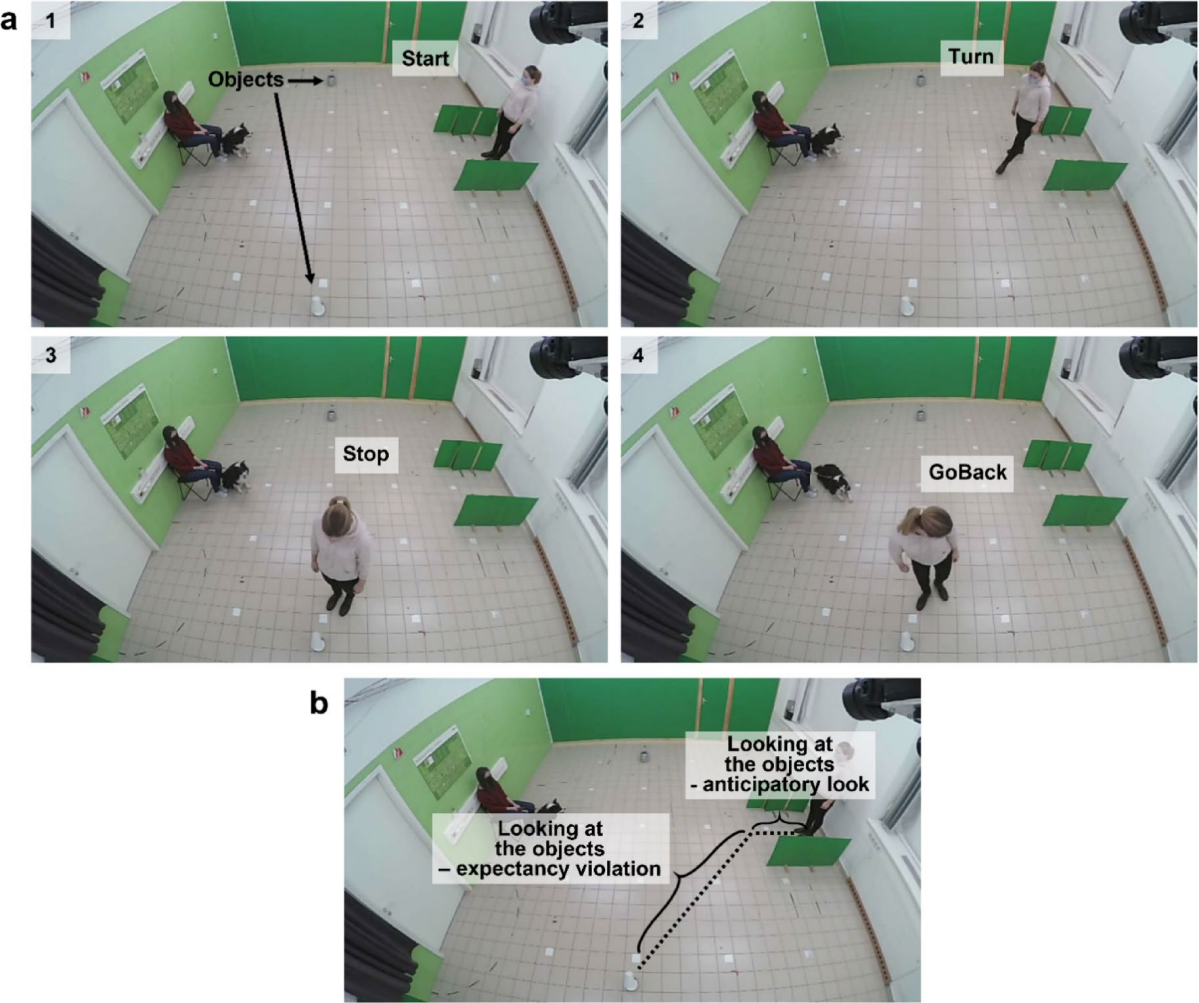
Procedure of the Demonstration and Test phases. (**a**) Steps of approaching the object(s): (1) After standing still for 5 s, the actor moved straight forward within the screens (Start), then (2) turned toward the object (Turn), and (3) stopped next to the object for 7 s (Stop), finally, (4) turned (GoBack) and went back to the starting position on the same route. (**b**) The parts of the route during which looking at the objects behaviour was analysed.

In the Ambiguous UMO group, we used two green screens made by cartonplast with wooden legs (130 cm length, 60 cm height) to cover the starting and ending position of the UMO during the Introduction phase (see Procedure) (Fig. 5). We used the same screens to make a corridor for the actors in the Demonstration phase, in all groups (Fig. 1), thus creating a physical barrier due to which the actor could only move straight forward until leaving this corridor. This way we could measure dogs’ anticipatory look after the actor started to move but before they turned toward either of the objects.

In the case of the Introduction phase of the Ambiguous group, we used a blue screen (125 cm x 100 cm with two bent sides of 125 cm x 70 cm) to cover subjects’ view of the room while E made noise both at the starting/ending position of the UMO (see Procedure).

### Procedure

We introduced dogs to the UMO or the human actor during the *Introduction phase*, which was followed by the *Demonstration* and then the *Test phase.* For a video about the procedure, see Supplementary Video 1.

#### Introduction phase

Before the dog entered the room, E placed a chair (and other apparatus if needed; see above) and the UMO in the room or the human stood in her starting position. The owner and the dog entered the room along E. The dog could explore the room while E gave the instructions to the owner. Following this, the owner sat on the chair and held the dog in front of him/her by the collar, and E left the room through door B. The actor started to move, and dogs observed the behaviour of the specific actor.

Ambiguous UMO group: In the case of inanimate motion, it is important for the onset of the motion to be facilitated by an animate agent or to be ambiguous, but while exploring the room, dogs could see the starting point of the UMO behind the cover. Thus, after the dog was held by the owner at the chair, E placed the blue screen in front of the dog to occlude its view of the room, and made noise behind the screens at both sides, making it possible to believe that an external force started the motion of the UMO, as well as stopping it (ambiguity).

The UMO displayed mechanistic motion; no cues associated with self-propelledness were presented. The start, stop and turning motions were covered by the green screens (Fig. 5). In the visible part of the path, the UMO moved with constant speed. After four rounds, the UMO stopped behind the screen at its starting position (Duration of the phase, mean (s) ± SD): Car, 122.0 ± 14.3; Ollie: 56.1 ± 4.1). E entered the room, covered the dog’s view again with the blue screen and made noise at the screens.

 Animate UMO group: The UMO displayed different animate motion cues that were previously described to elicit animacy perception, such as starting to move from rest (e.g.^1,28^), sudden changes in speed (e.g.^2,26^), and direction (e.g.^31^), having variability in the motion trajectory (e.g.^11^; for dogs, see also^22^). The UMO’s starting position was in the middle, next to the wall on the right side of the dog (Fig. 5). The UMO moved on various routes in the room with multiple accelerations and decelerations, stopped for 3–5 s (3 or 4 times) and then started to move again (Duration of the phase, mean (s) ± SD): Car, 102.7 ± 15.8; Ollie, 69.3 ± 0.8). At the end, the UMO stopped next to the wall, in front of the dog.

Interactive UMO group: Dogs observed the UMO as it engaged in interaction with an unfamiliar human (UH) (Duration of the phase, mean (s) ± SD): Car, 94.0 ± 3.7; Ollie, 66.6 ± 0.4). We aimed to introduce an interaction where the UMO does not move toward and picks up any objects, and dogs can recognize the interaction/commands from their everyday lives. The UMO’s starting position was in front of the dog, next to the wall opposite to the dog (Fig. 5). UH stood in the corner close to door B. The UMO started to move in the room in various routes with changing speed for about 15–25 s. UH walked to the side of the room opposite to the dog and her initial position. UH called the UMO by saying “UMO, come here!” while orienting toward the UMO. The UMO went to UH immediately, and UH petted and praised the UMO (as would do in case of a dog) for 5 s. UH gave the command “Stay” to the UMO, and then moved to the opposite side of the room. The UMO did not move. UH called the UMO again by saying “UMO, come here!”, while orienting toward the UMO. The UMO went to UH immediately, who petted and praised it for 5 s. UH and the UMO moved to the opposite side of the room together, while UH continuously encouraged the UMO to go with her. We repeated these steps (stay - come here - come with me) once more, at the end, UH and the UMO leaving the room together through door A.

Human actor group: The human stood at her starting position, in front of the dog, next to the wall opposite to the dog. She moved around the room on various routes with different speeds (Duration of the phase (Mean (s) ± SD): Female 1: 95.5 ± 0.8, Female 2: 93.3 ± 1.2), similarly as the UMO in the *Animate UMO group*. After 90 s elapsed (measured by the actor on a stopwatch), the actor left the room through door A.

At the end, the owner and the dog left the room through door A (the human actor went in the adjacent room to avoid meeting with the dog outside, and the the interactive UMO was placed inside this room by E).

#### Demonstration phase

Before the owner and the dog entered again, E placed in the room the screens, the actor and the two objects with equal distances from the actor and the dog (Fig. 1). The phone controlling the motion of the Sphero Ollie was placed next to door A (used by E during this and the next phase). The owner and the dog entered the room (with the dog on leash) along E. The owner sat on the chair immediately, without approaching any of the objects, and held the dog in front of them. Then, E left the room through door A. In the case of the Ollie robot as UMO, before she left the room, E started the program of the UMO on the phone.

The actor moved forward 1.3 m in the corridor made from the screens and turned toward the goal object after exiting the corridor (continuous motion). The actor stopped when reaching the object (0.5 m within the object), stayed there motionless for 7 s, and then moved back to its/her original position on the same route. Before the next trial, the actor spent 5 s at its starting position. This procedure was repeated 10 times (in the case of two dogs only 9 times, and in the case of two dogs 11 times) (Demonstration trials). After the last trial, E came back and accompanied the owner and the dog out of the room for a short break.

If the remote-controlled car’s battery discharged or the Ollie robot lost the Bluetooth signal during the phase, E entered the room on door A immediately, and the owner and the dog left the room for a short break. When the problem was solved, the trials continued (but see exclusions of subjects above).

#### Test phase

Before the owner and the dog came back, E switched the places of the objects; when the owner and the dog came back, the dog was on a short leash and was not allowed to approach the objects. The procedure was the same as described for the Demonstration phase. However, here the dog observed the actor moving three times to the goal object using a new route (Goal-consistent section), and three times approaching the non-goal object using the same route as in the Demonstration phase (Route-consistent section) (overall six Test trials). The order of the Goal-consistent and Route-consistent sections were counterbalanced between subjects.

### Behaviour and data analyses

We used Solomon Coder 19.08.02. (©András Péter) for coding the behaviour. We carried out the data analyses with R software version 4.3.2^32^ in RStudio version 2023.12.1 Build 402^33^. For the inter-coder reliability, we used a semi-random subsample (20% of dogs; counterbalanced between groups and actor type). We exported the specific sections of the coding sheets of both coders from the Solomon Coder and checked the correspondence between coders for all frames (5 frames per second). Inter-coder reliability was tested calculating Cohen’s kappas. Results indicated acceptable agreement between coders (mean κ ± SD: 0.675 ± 0.160; mean % of agreement ± SD: 97.5 ± 2.5).

We coded dogs’ looking duration at the goal and the other object in the Test phase, (1) from the moment the actor started to move until it turned toward one of the objects (i.e. when it moved straight forward) (Start-to-Turn), and (2) from the moment the actor turned toward one of the objects, until it left the object and started to go back to its original place (Turn-to-GoBack) (see Fig. 1):

In the first case (Start-to-Turn), we only included dogs’ looking duration at the goal and other object from the very first Test trial (Test trial 1), that is, after the places of the objects were switched, but the actor did not turn toward either of them on its route (anticipatory look).

In the latter analysis, we tested whether the actor changing the route or the approached object in Test trial 1 and 4 (the first demonstration of the specific action) influenced dogs’ looking behaviour toward the two objects (violation of expectation). Thus, we only used data from the two following trials (Test trial 2–3, and Test trial 5–6).

In both cases, instead of seconds we used percentages to standardize the data, due to the differences in the length of the trials. Data distribution was determined by using the check_distribution function (‘easystats’ package^34^), suggesting tweedie distribution for both models. Generalized linear mixed models (GLMMs) were fitted using the glmmTMB function (‘glmmTMB’ package^35^). To account for the large number of zero in the data, which we expected to include structural zeros due to dogs often not looking at the objects (looking at the actor or not looking at the demonstrated motion at all), we used the compare_performance function (‘easystats’ package^34^) to compared the tweedie model with a zero-inflated tweedie model in both cases, and chose the one with lower AIC value (ΔAIC ≥ 2). Based on the comparison, we used zero-inflated tweedie model in both cases (Start-to-Turn: AIC zero-inflated tweedie = 239.9, AIC tweedie = 277.4; Turn-to-GoBack: AIC zero-inflated tweedie = 853.9, AIC tweedie = 861.6). Regarding the final models, the zero-inflation component of the models estimated that approximately 80.8% of data points involved structural zeros in the case of the Start-to-Turn model, and 67.7% of data points in the Turn-to-GoBack model. For details about model fit, see Supplementary Information 1.

In the Start-to-Turn model (anticipatory look), we analysed whether there were differences between the four groups in looking at the goal and at the other object (two-way interaction of group and object).

In the Turn-to-GoBack model (violation of expectation), we analysed whether there were differences between the four groups in looking at the goal and the other object, irrespectively of the section (two-way interaction of group and object), and whether there were differences between the groups in looking at objects in general in the two sections (two-way interaction of group and section). We also included in the model whether the actor approaching the goal or other object in the specific trial influenced their looking duration toward the Goal and Other objects (two-way interaction of object and section). We further tested whether the first change in the Test phase (route or goal) had an effect on the percentage of looking duration at the objects.

In both cases, we used the drop1 function (‘stats’ package) for backward model selection. For the selection, we used likelihood ratio test (LRT), the non-significant variables were excluded from our model step-by-step (results of LRT are reported before exclusion). For the significant variables in the final model, we carried out pairwise comparison (Tukey adjustment; ‘emmeans’ package^36^) and we report the contrast estimates (β ± SE).

## Supplementary Information

Below is the link to the electronic supplementary material.

Supplementary Material 1

Supplementary Material 2

Supplementary Material 3

## Data Availability

All data are available as Supplementary material.
