# Supplementary material for: Perception of animacy leads to expectation of goal-directed behaviour in dogs
Source: Sci Rep. 2026 Jan 13;16:1758. doi: 10.1038/s41598-025-26837-w (PMC12804912; doi:10.1038/s41598-025-26837-w)
Supplement: Supplementary file 3 — Supplementary Material 3 [file 41598_2025_26837_MOESM3_ESM.pdf]

Supplementary information for:

Perception of animacy leads to expectation of goal-directed behaviour in dogs

By Z. Gedai, Á. Miklósi, & J. Abdai

In *Scientific Reports*

Assessing model fit was carried out using DHARMa package [1].

I. Model fit for: *Looking at the objects in Test trial 1 from Start to Turn (anticipatory look)*

The starting model was chosen by using compare_performance function ('easystats' package [2]), suggesting tweedie with zero-inflation (ZI) (based on AIC) (Table S1).

Table S1. Comparison of the starting models for anticipatory looking, using tweedie distribution and tweedie with ZI.

Name	Model	AIC (weights)	AICc (weights)	BIC (weights)	R2 (marg.)	RMSE	Sigma
tweedie	glmmTMB	277.4 (<.001)	279.2 (<.001)	311.2 (<.001)	1.000	4.849	13.844
ZI_tweedie	glmmTMB	239.9 (>.999)	242.1 (>.999)	276.8 (>.999)	1.000	4.912	0.643

For the performance of the final model, see Table S2 and Figure S1.

Table S2. Performance of the final model for anticipatory look.

Name	AIC	AICc	BIC	R2 (marg.)	RMSE	Sigma
ZI_tweedie_final	231.290	231.839	249.741	1.000	4.879	0.714

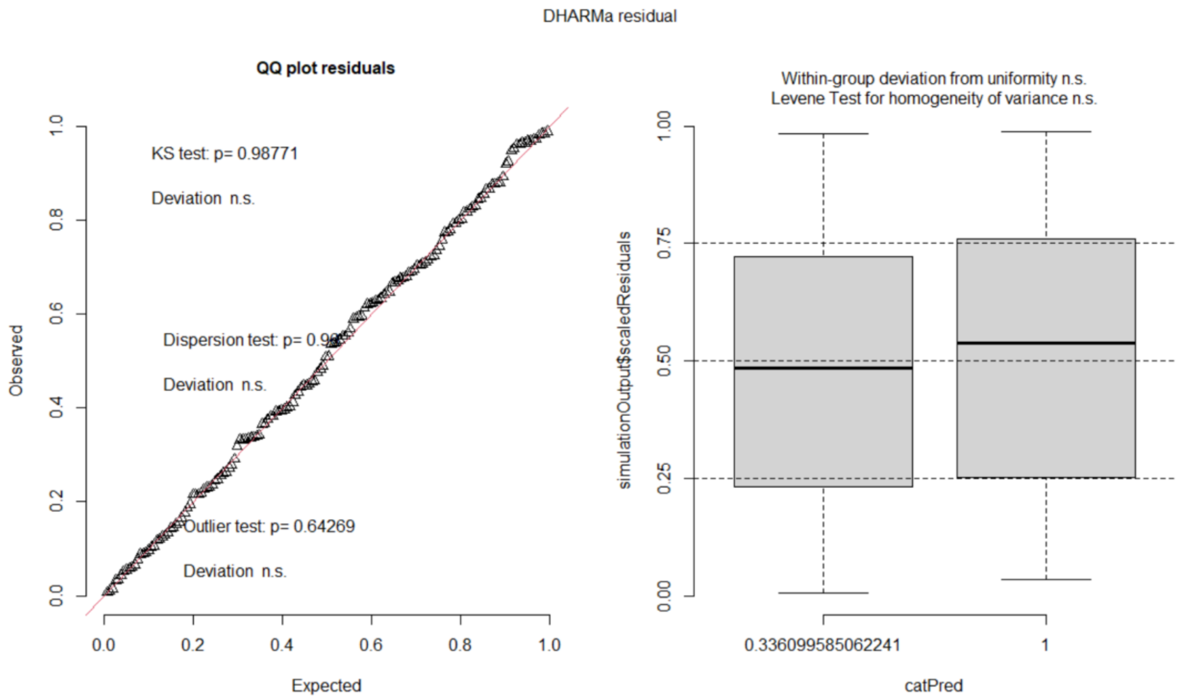

Figure S1. Model fit of the final anticipatory look model, using DHARMa package [1].

II. Model fit for: *Looking at the objects in the Test phase from Turn to GoBack (violation of expectation)*

The starting model was chosen by using compare_performance function [2], suggesting tweedie with ZI (based on AIC) (Table S3).

Table S3. Comparison of the starting models for expectancy violation, using tweedie distribution and tweedie with ZI.

Name	Model	AIC (weights)	AICc (weights)	BIC (weights)	R2 (cond.)	R2 (marg.)	RMSE	Sigma
tweedie	glmmTMB	861.6 (0.020)	862.6 (0.022)	937.5 (0.163)	0.196	0.130	0.801	4.150
ZI_tweedie	glmmTMB	853.9 (0.980)	855.0 (0.978)	934.2 (0.837)	0.440	0.220	0.786	0.869

After backward model selection, keeping only significant variables, we found that the model fit had some problems (using DHARMa package [1]): KS test, p = 0.02, and significant within-group deviation from uniformity (no other significant deviation) (Figure S2).

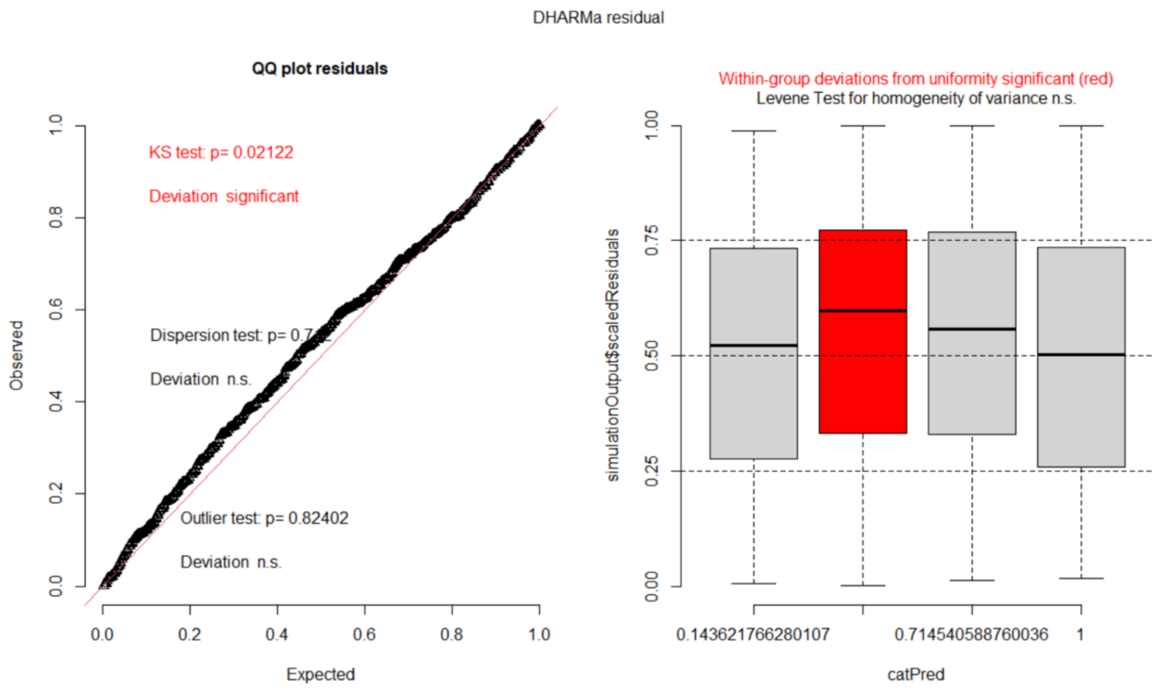

Figure S2. Model fit of the final expectancy violation model, using DHARMa package [1]. Model fit indicate some deviations.

Due to this, we ran backward model selection using tweedie distribution without ZI (as tweedie already accounts for zeros). We had the same significant two-way interaction:

- Change: $\chi^2_1 = 0.002$, p = 0.967
- Group x Section: $\chi^2_3 = 0.282$, p =0.963
- Group x Object: $\chi^2_3 = 1.211$, p =0.750
- Group: $\chi^2_3 = 1.180$, p =0.758
- ***Object x Section:*** $\boldsymbol{\chi^2_1 = 11.605}$***, p < 0.001***
  - *Goal vs other: Goal-consistent, β ± SE = -0.596 ± 0.406, p = 0.143*
    Route-consistent, β ± SE = 1.255 ± 0.354, p < 0.001
  - *Goal- vs Route-consistent:* ***Goal, β ± SE = -1.584 ± 0.373, p < 0.001***
    Other, β ± SE = 0.266 ± 0.388, p = 0.493

We used the compare_performance function [2] again to compare the final models with and without ZI, which indicated negligible difference (Table S4).

Table S4. Comparison of the final models for expectancy violation, using tweedie distributio n and tweedie with ZI.

Name	Model	AIC (weights)	AICc (weights)	BIC (weights)	R2 (cond.)	R2 (marg.)	RMS E	Sigma
ZI_tweedie _final	glmmTMB	843.3 (0.622)	843.6 (0.616)	879.0 (0.150)	0.340	0.165	0.790	1.526
tweedie_ final	glmmTMB	844.3 (0.378)	844.5 (0.384)	875.6 (0.850)	0.193	0.116	0.802	4.179

For model fit of the final model with tweedie distribution, but without ZI, see Figure S3.

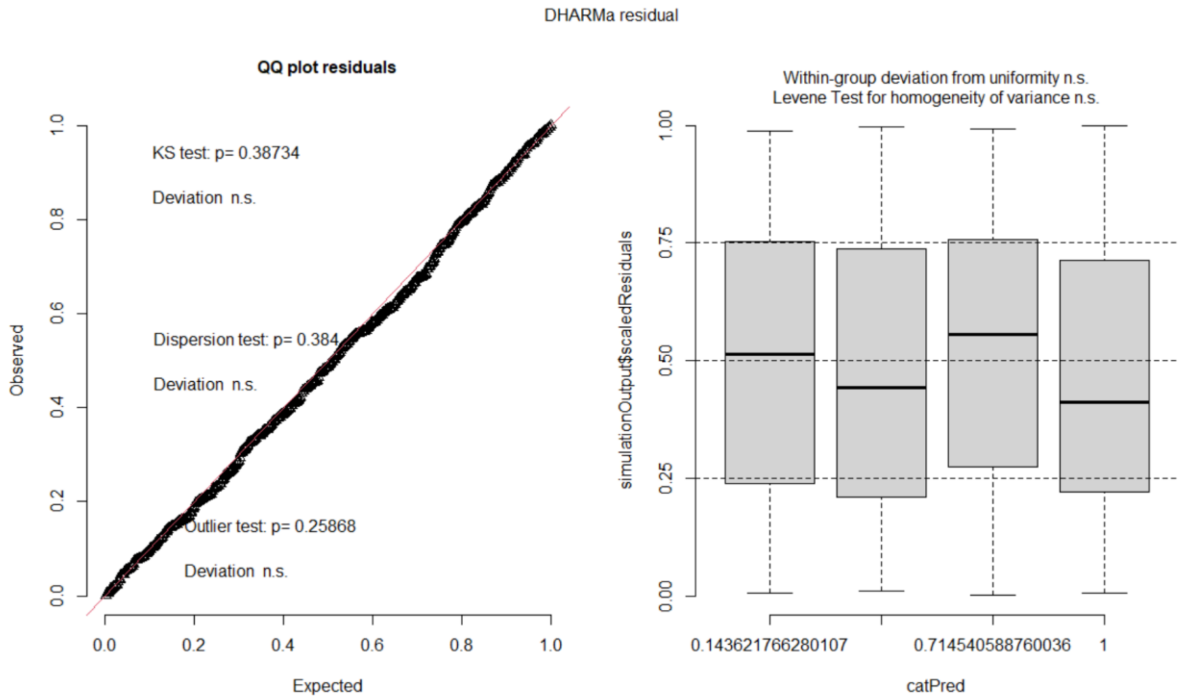

Figure S3. Model fit of the final expectancy violation model when fitted with tweedie distribution (without ZI).

However, we tested whether zero-inflation is meaningful: based on the result, zero-inflation probability was estimated at 0.68 (95% CI: 0.48–0.83), indicating a substantial structural zero component (at least about 50% of observations were expected to be “extra” zeros, beyond the tweedie). Thus, despite the slight issues with the model fit of the original model, we decided to keep the tweedie distribution with ZI.

References

1. Hartig, F. DHARMa: Residual diagnostics for hierarchical (multi-level / mixed) regression models. Preprint at https://CRAN.R-project.org/package=DHARMa (2022)

2. Lüdecke, D. *et al.* easystats: Framework for easy statistical modeling, visualization, and reporting. Preprint at https://easystats.github.io/easystats/ (2022)
